# Temporomandibular Joint Minimally Invasive Procedures in the Pediatric Population: A Prospective Study

**DOI:** 10.3390/jcm13030672

**Published:** 2024-01-24

**Authors:** David Faustino Ângelo, Cláudia Sacramento Lopes, David Sanz, Maria Cristina Faria-Teixeira, Rute Marques, Francesco Maffia, Henrique José Cardoso

**Affiliations:** 1 Instituto Português da Face, 1500-493 Lisboa, Portugal; david.sanz@ipface.pt (D.S.); rute.marques@ipface.pt (R.M.); henrique.cardoso@ipface.pt (H.J.C.); 2 Centre for Rapid and Sustainable Product Development, Polytechnic Institute of Leiria, 2430-028 Marinha Grande, Portugal; 3 Faculty of Medicine, Lisboa University, 1649-028 Lisboa, Portugal; claudia-lopes@campus.ul.pt (C.S.L.); cristina.vft@gmail.com (M.C.F.-T.); 4 Maxillofacial Surgery Unit, Department of Neurosciences, Reproductive and Odontostomatological Sciences, University of Naples “Federico II”, Via Sergio Pansini 5, 80131 Naples, Italy

**Keywords:** minimally invasive surgical procedures, temporomandibular joint disorders, arthroscopy, TMJ arthrocentesis, pediatrics

## Abstract

Over recent years, temporomandibular joint (TMJ) minimally invasive procedures, such as arthrocentesis and arthroscopy, have been appointed as an initial TMJ intra-articular treatment. Both procedures present safe and effective clinical results in managing temporomandibular disorders (TMD) by reducing pain and improving mouth opening. The use of these techniques in adults is validated in the literature. However, data on the safety and effectiveness of minimally invasive TMJ interventions in pediatric patients are scarce. This study aims to investigate the effectiveness of TMJ arthrocentesis and arthroscopy in the pediatric population. A prospective study was conducted at Instituto Português da Face (IPF) in Lisbon, Portugal, including patients treated for TMD from 1 June 2019 to 30 June 2023. In the present study, 26 patients (17 female and 9 male) were included, representing a total of 48 joints operated. A statistically significant reduction was observed in the primary outcome, TMJ pain, from 3.93 ± 2.80 preoperatively (mean ± SD) to 0.50 ± 1.53 (mean ± SD) postoperatively (*p* < 0.05). An improvement in the secondary outcome, maximum mouth opening, from 36.92 ± 8.79 preoperatively to 42.96 ± 5.07 postoperatively, was observed (*p* < 0.05). The overall success rate was 84.62%. This prospective study showed that TMJ arthrocentesis and arthroscopy appear to benefit pediatric patients with TMD, significantly lowering pain and improving MMO without relevant postoperative complications.

## 1. Introduction

Over recent years, temporomandibular joint (TMJ) minimally invasive procedures have been appointed as an alternative for conservative treatment failure in cases of severe acute pain or chronic pain, inflammation, and/or degeneration disease, namely in arthrogenous temporomandibular disorders (TMD) [1]. The main advantages of these techniques are: minimal distress and reduced invasiveness to the patient, reduced risk of complications [2,3], faster recovery, faster results, minimal cosmetic deformity, low emotional impact, improved quality of life [4], and high satisfaction. Although they present more risks/complications associated with operator errors and anesthetic effects, TMJ arthrocentesis and arthroscopy are generally safe procedures with low incidence of major complications [2,5,6,7]. The level of safety is closely tied to the surgeon’s expertise. In the rare occurrence of complications, they are usually temporary, and spontaneous remission occurs in most cases without needing treatment. The surgeon must exercise caution to prevent potential risks such as vascular and nerve injuries, brain damage, particularly by avoiding perforation of the glenoid fossa, or ear lesions [8]. These treatment options, especially TMJ arthrocentesis and arthroscopy, showed faster, more effective clinical results and long-term results over conservative approaches in managing arthrogenous TMD to reduce pain and improve mouth opening [9,10,11,12]. TMJ double-puncture arthrocentesis is a simple but technically challenging, office-based procedure performed most of the time under local anesthesia. This technique’s main goals are removing chemical inflammatory mediators and changes in intra-articular pressure through joint lavage with the possibility of depositing therapeutic substances. TMJ arthroscopy is mainly performed under general anesthesia. Through this approach, it is possible to visualize the upper compartment, perform diagnosis, lavage, and biopsies, remove adhesions, treat synovial inflammation, and perform disc repositioning [13,14]. Level 1 TMJ arthroscopy is the most basic approach, with observation of the upper compartment, lysis, and lavage and the possibility of depositing therapeutic substances. More advanced arthroscopic techniques are used in level 2–3 arthroscopy. It is recognized to be effective in reducing pain and restoring mandibular function, with minimal morbidity. The use of these techniques in adults is well-validated in the literature [3,9,15,16]. However, data on the effectiveness and safety of minimally invasive TMJ surgery in pediatric patients are limited. Thus, it is crucial to investigate the results of minimally invasive interventions in this age group. In children, the available data focus on conservative treatments: patient education on the disease and its pathology; behavioral therapy; cognitive behavioral therapy (CBT); physical therapy, which might include jaw exercises, transcutaneous electrical nerve stimulation (TENS), ultrasound, massage, TMJ distraction and mobilization, thermotherapy, and coolant therapy; pharmacological therapy; and occlusal splints, to provide orthopedic stability to the TMJ [17]. However, these treatments also have disadvantages. An occlusal splint can be complex, especially in mixed dentition cases [18]. Moreover, it was also demonstrated that an occlusal splint combined with physiotherapy can induce craniovertebral and craniomandibular changes in TMD patients, namely the vertical and sagittal position of the mandible and the width of the functional space between C1 and C2 [19]. Pharmacotherapy should be managed carefully, especially in the pediatric population [20]. Clinicians seem reluctant to adopt minimally invasive approaches in infants and adolescents, since there is not enough evidence to perform these procedures safely and confidently. This prospective study intends to improve knowledge on this topic, demonstrating the results of TMJ arthrocentesis and arthroscopy in the pediatric population with arthrogenous TMD. The primary outcome is TMJ pain, the secondary outcomes are: the maximum mouth opening (MMO), myalgia degree, and the presence of joint clicking.

## 2. Materials and Methods

### 2.1. Study Design

This prospective study was conducted at Instituto Português da Face (IPF) in Lisbon, Portugal, and included patients treated for TMD from 1 June 2019 to 30 June 2023. The Ethics Committee of Instituto Português da Face approved this investigation (PT/IPFace/RCT/1906/08 on 13 May 2019), and all enrolled patients and their legal guardians gave their informed consent in writing, following current legislation. The study follows the guidelines of the Declaration of Helsinki. Patient data were registered in the EUROTMJ database ((https://eurotmj.org), first access for this study 1 June 2019), scrubbed of any personally identifying parameters, and given a random ID number.

The inclusion criteria were: (1) age < 18 years old; (2) conservative treatment without any improvement for at least three months; (3) clinical and imaging (magnetic resonance imaging (MRI) and/or computed tomography (CT)) diagnosis of arthrogenous disorder disc displacement with reduction (DDwR); DDwR with intermittent locking, disc displacement without reduction (DDwoR) with/without limited opening, degenerative joint disease; (4) Dimitroulis classification between 2 and 4 where most components of the joint were salvageable [21]; (5) one-month minimum follow-up time. Exclusion criteria: (1) previous TMJ surgical intervention; (2) severe medical problems; or (3) impaired cognitive capacity.

The same TMJ surgeon examined and treated all patients (David Faustino Ângelo, M.D., Ph.D.). Parafunctional habits such as awake and night bruxism were assessed. All sleep and awake bruxism habits were considered by parents for 2 weeks in accordance with international recommendations [22]. During oral cavity examination, particular attention was given to mucosal signs and muscular activity related to bruxism. A multimodal conservative treatment approach was performed in all patients for at least 3 months, including: behavioral reeducation, physiotherapy, and, in specific cases of acute pain, pharmacotherapy (non-steroidal anti-inflammatory drugs and/or muscle relaxants) adapted to the age and body weight.

The primary outcome was TMJ pain (arthralgia) reduction. Arthralgia was reported in case of: (1) history of pain in the TMJ area and (2) pain on palpation of the lateral pole or around the lateral pole, or pain during maximum unassisted or assisted opening, right or left lateral movements, or protrusive movements. TMJ arthralgia was scaled through a visual analog scale (VAS, 0–10, with 0 being no pain and 10 being maximum insupportable pain). To facilitate the use of the VAS, the standard scale was enriched with colors and smileys to improve interpretation by the pediatric population [23].

The secondary outcomes were MMO improvement, myalgia degree, and the presence of joint clicking. MMO (mm) was measured between the incisors using a certified ruler. Myalgia was assessed according to a clinical history positive for: (1) during the past 30 days, pain in the jaw, in front of the ear, with examiner confirmation of pain location in masticatory muscles, and (2) pain modified with jaw movement, function, or parafunction and a positive clinical evaluation for palpation pressure (5 s/1 kg pressure) in masseter and temporalis muscles as defined in DC/TMD [24]. Myalgia was graded accordingly with pain intensity in the masseter and temporalis muscles: 0 = no pain/pressure only; 1 = mild pain; 2 = moderate pain; 3 = severe pain [25].

Joint noise (click or crepitus) was registered with a positive history of TMJ noises during the 30 days before the examination or by detecting any joint noise with jaw movements during the clinical examination.

All assessments were conducted preoperatively, 1–2 months before the TMJ intervention (T0), and postoperatively (T1) at various intervals, including 1 month, 3 months, 6 months, 1 year, and annually after that. The success criteria were determined based on two categories for classifying TMJ pain: favorable/good if VAS ≤ 2 and unfavorable/failure if VAS > 2. Regarding MMO, success was defined with a cutoff of MMO ≥ 35 mm (considered good if ≥35 mm and acceptable if between ≥30 mm and <35 mm), while failure was defined for MMO < 30 mm in the postoperative assessment. The success rate of surgery was graded as good, acceptable, or failure in accordance with Table 1 as described by [26].

The decision on the type of minimally invasive TMJ procedure to be carried out was based on Dimitroulis’ classification: category 2—TMJ minor changes; 3—TMJ moderate changes; 4—TMJ severe changes [21]. All patients classified with Dimitroulis 2 had an indication for TMJ arthrocentesis, and Dimitroulis 3 and 4 had an indication for TMJ arthroscopy.

### 2.2. Minimally Invasive TMJ Surgery

Patients experiencing myalgia grades 2 and 3 received additional treatment for the masticatory muscles before the minimally invasive TMJ intervention. This involved the administration of either 155U or 195U of incobotulinum toxin A (Xeomin^®^-Merz, Frankfurt, Germany), with an equal distribution in the right and left temporal and masseter muscles. This preoperative intervention occurred 15 days before the scheduled surgical procedure [27]. Following the surgery, patients were instructed to follow a soft diet for three days and engage in five physiotherapy and three speech sessions, starting three to five days after the intervention.

#### 2.2.1. Double-Puncture TMJ Arthrocentesis

All the details of this procedure have been recently published [3]. Briefly, asepsis was performed with betadine, and a sterile drape was placed. Local anesthesia was carried out with lidocaine and adrenaline. The first puncture was performed through careful palpation of the lateral rim of the glenoid fossa. A 5 cc syringe was prepared with a 1.8 cc dilution with lidocaine and adrenaline (1:80.000) and 3 cc of Ringer lactate. A 21 G needle coupled with a 5 cc syringe was gently introduced. After the needle tip contacted the posterior slope of the eminence of the upper joint compartment, it was verticalized to reach the upper compartment. Validation with a successful pumping action and the inflow and outflow of fluids in the joint was performed. The second puncture was performed anteriorly with a 21 G needle. After an effective circuit was completed, a lavage with ≥100 mL of Ringer lactate solution was performed. After the lavage, the joint was supplemented with 1.5 mL of low molecular weight hyaluronic acid (Suplasyn^®^-Viatris, Canonsburg, Pensilvânia, EUA, 20 mg/mL).

#### 2.2.2. TMJ Arthroscopy

For the TMJ arthroscopy procedure, a 1.9 mm arthroscope was utilized with a video system (Stryker, San Jose, CA, USA) and a 2.8 mm outer protective cannula, as previously outlined [16]. Following the principles of the Holmlund–Hellsing (H-H) line, the authors implemented a traditional puncture at a point situated 10 mm anterior and 2 mm below for TMJ arthroscopy level 1. The arthroscope was inserted into the superior joint space. Following that, a second puncture was performed using a 21 G needle, positioned 30 mm anterior and 7 mm below the H-H line, to irrigate the joint with 250–300 mL Ringer solution. In the context of level 2 TMJ arthroscopy, the second puncture was carried out with a 2.8 mm outer protective cannula with a sharp trocar, extending into the joint. This cannula was a conduit for (1) the ReFlex Ultra 45 Plasma Wand system for intra-articular coblation and/or (2) intrasynovial medication via a 22 G long spinal needle. The determination of the intervention level during TMJ arthroscopy was guided by specific criteria: level 1 if the roofing percentage was 100% without synovitis; level 2 if the roofing percentage exceeded 50% and/or synovitis was present. Additionally, a supplementary deposition of 1.5 mL hyaluronic acid was administered. Postoperatively, patients received non-steroidal anti-inflammatory drugs for 5 days. In this study, no postoperative antibiotic was given.

### 2.3. Statistical Analysis

The data underwent analysis through GraphPad Prism (v10.1) and SPSS (v26) software. Variables were presented as the mean (±standard deviation (SD)) or percentage (%). Students’ paired *t*-tests were employed for variables exhibiting a normal distribution, while the Wilcoxon signed-rank test was utilized for variables lacking a normal distribution. The comparison between TMJ arthrocentesis and arthroscopy was assessed using a Mann–Whitney test. Statistical significance was established at *p* < 0.05.

## 3. Results

A total of 26 patients (17 female and 9 male) were included, representing a total of 48 joints operated. The mean age was 14.81 ± 2.30 (mean ± SD) years. Awake and sleep bruxism (parafunctional habits) were identified in 13 (50%) and 7 (26.9%) patients, respectively. Additionally, past orofacial treatments and associated events were identified: eight (30.7%) patients had undergone previous orthodontic treatment, two (7.69%) were subjected to wisdom teeth removal, and two (7.69%) suffered facial trauma. Asthma was the most common comorbidity in the young population of this study (15.39%, n = 4 patients). In a total of 52 joints, 48 joints were diagnosed with arthrogenous TMD: (1) disc dislocation without reduction (DDwoR) with arthralgia (20.83%, n = 10 joints), (2) disc displacement with reduction (DDwR) (18.75%, n = 9 joints); (3) arthralgia (16.67%, n = 8 joints); (4) DDwR with arthralgia (14.58%, n = 7 joints). Eighteen (69.23%) patients presented concomitant masticatory myalgia: level I—one (11.54%); level II—seven (26.92%); level III—eight (30.77%) (Table 2). Seven joints presented concomitant osteoarthrosis (OA) (14.58%, n = 7 joints). Thirty-two joints underwent TMJ arthrocentesis, and sixteen joints underwent TMJ arthroscopy.

**Figure 1:**
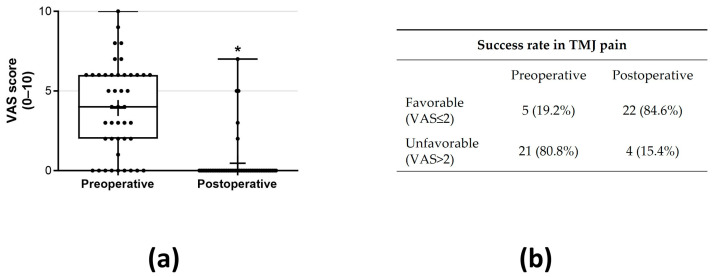
The statistical analysis (**a**) and success rate (**b**) for VAS were compared between preoperative and postoperative VAS outcomes. In the box-and-whisker graph, the horizontal line represents the median, the edges of the box indicate the 25th and 75th percentiles and the whiskers encompass the smallest and largest values within 1.5 box lengths from the box. * *p* < 0.05 indicates significant deviation when compared to preoperative VAS results.

**Figure 2:**
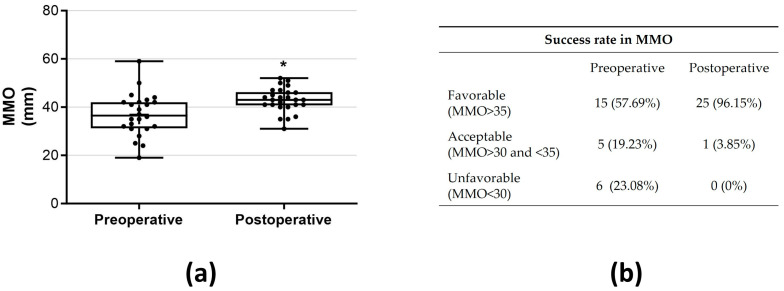
The outcomes of the statistical tests (**a**) and the success rate (**b**) for MMO were evaluated by comparing preoperative and postoperative MMO results. In the box-and-whisker graph, the horizontal line represents the median, the box edges indicate the 25th and 75th percentiles and the whiskers depict the smallest and largest values. All data points are represented with circles. * *p* < 0.05 denotes significant differences when compared to preoperative MMO results.

**Figure 3:**
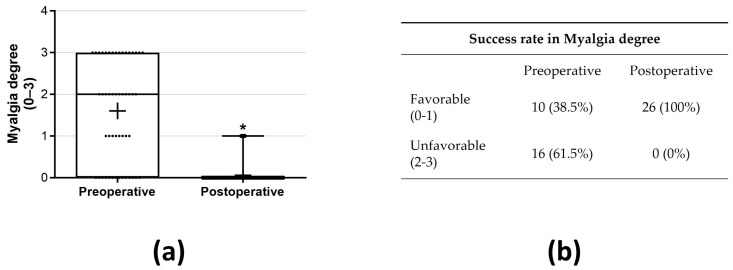
The results of the statistical tests (**a**) and the success rate (**b**) for myalgia degree were examined by comparing preoperative and postoperative myalgia degree outcomes. In the box-and-whisker graph, the horizontal line indicates the median, the box edges represent the 25th and 75th percentiles, and the whiskers illustrate the smallest and largest values within 1.5 box lengths from the box. * *p* < 0.05.

**Figure 4:**
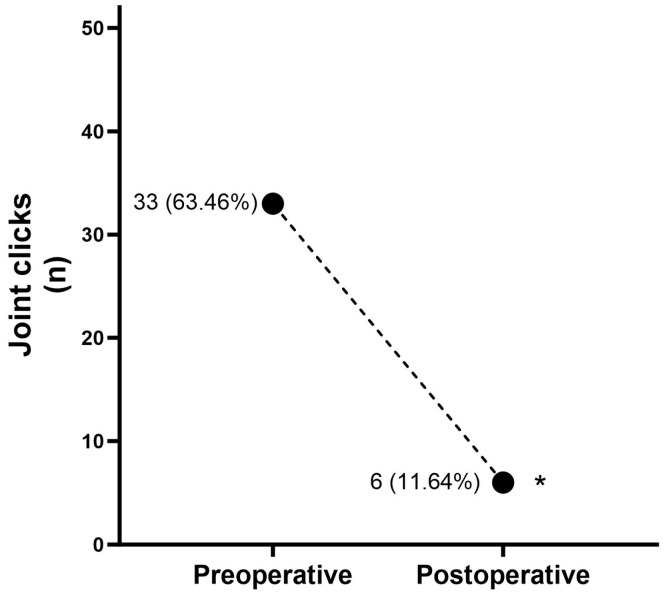
The results of the statistical tests comparing preoperative and postoperative joint click results * *p* < 0.05 when compared to preoperative joint clicks.

A statistically significant decrease was noted in the primary outcome, TMJ pain, decreasing from 3.93 ± 2.80 preoperatively (mean ± SD) to 0.50 ± 1.53 (mean ± SD) postoperatively (*p* < 0.05, r = 0.46—moderate effect; Figure 1a). The proportion of patients exhibiting a favorable outcome with reduced pain was 84.6% (Figure 1b). Preoperatively, 21 (80.8%) patients presented pain > 2 (VAS 0–10), while postoperatively 4 patients (15.4%) were classified as unfavorable (Figure 1b). An MMO improvement was observed from 36.92 ± 8.79 mm preoperatively to 42.96 ± 5.07 mm postoperatively (*p* < 0.05, r = 41—moderate effect; Figure 2a). Following the surgical procedure, MMO ≥ 35 mm was observed in 25 patients (96.15%), and none of the patients failed to achieve an opening of less than 30 mm postintervention (Figure 2b). A significant decrease in myalgia severity was observed, with a preoperative mean ± SD of 1.60 ± 1.21 compared to 0.06 ± 0.24 postoperatively (*p* < 0.05, r = 0.53—large effect; Figure 3a). Furthermore, all patients (100%) exhibited either no myalgia or a low grade (0–1) postoperatively (Figure 3b). Clicks were identified in 33 (63.46%) joints preoperatively, while postoperatively, this number reduced significantly to 6 joints (11.64%) (*p* < 0.05; Figure 4).

Considering Table 1, showing the success rate, a single minimally invasive TMJ procedure was considered successful in 20 patients (75.92%) and failed in 6 patients (23.08%) (Table 3). Two patients presented with new arthralgia and were managed with TMJ arthrocentesis. After the additional intervention, the success rate increased to 84.62% (n = 22).

VAS pain scores and MMO were also evaluated regarding the minimally invasive TMJ procedure type performed (Table 4). No differences were shown in these parameters when comparing the techniques used. No surgical or wound-healing complications were observed in any patient.

## 4. Discussion

This prospective study showed that TMJ arthrocentesis and arthroscopy appear to benefit pediatric patients with TMD, significantly lowering pain, improving MMO, and reducing clicks, without irreversible postoperative complications.

The study’s success with one minimally invasive TMJ procedure was 76%. In two patients, it was necessary to perform an additional arthrocentesis to manage TMJ arthralgia. Afterward, the success rate increased to 85%. The overall success of these minimally invasive surgeries in pediatrics is similar to adults. Recently, in two studies carried out by our research group, demonstrated a success rate of 76% for arthrocentesis and 69% for arthroscopy in adult patients (one single intervention) [3,16]. Also, several studies have shown a success rate for TMJ arthrocentesis and arthroscopy around 70% and 90% [28,29]. Nitzan et al. [30] reported a more than 81% success rate for TMJ arthrocentesis. Sembronio et al. [31] disclosed an overall success rate of 72.7% in closed-lock patients, reporting a higher rate, 87.5%, in patients with acute symptoms [32]. Fifty patients with degenerative pathology of the TMJ reported a 73% global success rate for arthroscopy, according to a study by Indresano [33]. Fridrich and Zeitler [15] reported an 82% success rate for TMJ arthroscopy and 75% for arthrocentesis. No significant difference was found between these interventions.

Our study found statistically reduced pain (VAS ≤ 2) in 84% of child patients. No differences in pain reduction were demonstrated between TMJ arthrocentesis and arthroscopy. Equally, in a retrospective study including 23 pediatric patients who underwent arthroscopy, the VAS scores improved 25–26% in the short and long term [32]. Similarly, we demonstrated a reduction of pain in 86% of adult patients submitted to TMJ arthrocentesis and arthroscopy [3,16]. Also, in a sample of 50 adults (78 joints) submitted to arthroscopy, Indresano et al. [33] verified a reduction in pain in 70% of patients. Alpaslan et al. [28] evaluated patients with degenerative pathology of the TMJ for a follow-up period of 22 months (range: 3–60 months) after arthrocentesis, observing significantly reduced pain and dysfunction. A retrospective study that analyzed 20 patients showed a reduction of pain of 4.56 ± 1.74 (VAS) for the arthrocentesis group and 2.5 ± 2.2 (VAS) for the arthroscopy group [34]. These results suggest that applying these techniques is equally effective in reducing pain in pediatric and adult patients.

In our study, significant improvement in MMO was observed. The mean preoperative MMO was 36.92 ± 8.79 mm. MMO improved to 42.96 ± 5.07 mm postoperatively, and 25 patients (96.15%) had MMO ≥ 35 mm. In another study covering 23 pediatric patients, the results of mouth opening increased by 5.4 and 8.2 mm in the short-term and long-term, respectively [32]. Perceived jaw dysfunction improved significantly, with an average improvement of 23.8% in the short-term and 19.2% in the long-term. Equally, in the adult population, a study following arthrocentesis revealed that patients had a significant increase in mouth opening, from 24.1 ± 5.6 mm to 42.7 ± 4 mm [30]. A 6-year retrospective study in patients submitted to TMJ arthroscopy reveals that 56% of patients experienced an excellent range of motion, which accounts for a vertical mouth opening of 40 mm, and in 7 of 12 centers, more than 70% of patients reported excellent results; in the 3 centers reporting less than 20% excellent results, nearly 80% of the results were reported as good (vertical mouth opening between 30 and 40 mm) [35]. In another retrospective study analyzing 20 patients, the mean MMO was 26.56 ± 2.74 mm and 30.25 ± 3.73 mm before TMJ arthrocentesis and arthroscopy, respectively. Postoperatively, MMO was 39.56 ± 3.36 mm in the arthrocentesis group and 36.88 ± 7.43 mm in the arthroscopy group [34]. Murakami [32] also described that TMJ arthrocentesis and arthroscopy were equally effective in treating closed lock of the TMJ, but they concluded that arthrocentesis was a better option in acute closed lock. However, Goudot et al. [36] stated that TMJ arthrocentesis and arthroscopy were both valid treatment options for TMD, but arthroscopy was more successful in improving mouth opening. The results obtained in this study in pediatric patients with improved mouth opening after arthrocentesis and arthroscopy are comparable to those obtained in previous studies in adults.

In adults, minimally invasive techniques had rare complications. In TMJ arthrocentesis, complications typically arise temporarily due to the anesthetic effect or the soft tissue swelling resulting from fluid extravasation during the irrigation procedure [5,6]. These issues can be effectively addressed through outpatient management. Also, complications related to the arthroscopic technique are not numerous. They are mainly represented by extravasation of the fluids used for irrigation with the possibility of pharyngeal edema, intra-articular bleeding during myotomy in the anterior recess, iatrogenic joint damage (disc perforations, fragmentation of the articular eminence, excessive synovial fibrillation), and damage to the external auditory canal or middle ear [2,7]. Despite being minimally invasive, these procedures performed in children can also have complications that could affect the normal development of the temporomandibular joint. The TMJ goes through two major periods of increased growth: between 5 and 10 years and between 10 and 15 years [37]. Therefore, these are delicate age groups. In the case of juvenile patients below 20 years old, anterior disc displacement without reduction was linked to a reduction in condylar height, attributed to either condylar resorption or the cessation of condylar growth [38]. In the same age group, Xie et al. [39] reported substantial differences in the height of the affected condyle and observed progressive mandibular asymmetry over a 12-month follow-up period. This robustly supports the notion that disc displacement may contribute to condylar resorption. Arthroscopy with the goal of disc repositioning could help prevent the condyle’s ongoing resorption. Further studies on this phenomenon are necessary to determine how disc repositioning influences condylar re-modeling and growth [40].

Regarding joint clicking, a significant decrease was recorded in 33 (63.46%) joints preoperatively while, postoperatively, it was recorded in 6 joints (11.64%). In a retrospective study by Choi et al. [40], of the 23 pediatric joints that presented noise, 14 (56%) had resolved after arthroscopy. Similarly, in adults in a retrospective study, 13 patients had TMJ clicks, and 12 patients (92.3%) no longer had this symptom after 4 months following arthrocentesis [41].

Preoperatively, ~62% of the patients presented myalgia >1. Postoperatively, no patient had high levels of myalgia. This variable is an important indicator because it is closely linked to pain levels [3]. Moreover, it is very susceptible to external factors, such as parafunctional activities with no resolution, psychological factors, stress, and anxiety, or even associated with other diseases, like fibromyalgia. Given the failure of conservative treatments such as physiotherapy to reduce myalgia, we had recourse to the use of botulinum toxin. Botulinum toxin in pediatric patients is widely used in the neurological [42] and orthopedic [43] fields. Although this procedure is safe and well-tolerated, it has complications related to possible changes in bone density and growth interference [44]. In our experience, no short-term nor long-term complications were recorded.

Despite most TMDs being diagnosed around the ages of 30–40 [45], a study of 4724 children aged 5 to 17 showed that 25% had symptoms compatible with TMD [46]. Some etiologic factors are mentioned as a reason for the TMD development in the pediatric population: macrotrauma, which frequently occurs in childhood (unilateral and bilateral intracapsular or subcondylar fractures are the most common mandibular fractures in children); microtrauma from parafunctional habits, which overload the joint and promote the development of changes within the joint; psychosocial factors, like somatization, anxiety, and stress, obsessive–compulsive personality types; and systemic and pathologic factors, which include connective tissue diseases, joint hypermobility, genetic susceptibility, and hormonal fluctuations [47]. In the study conducted by Mehdipour et al. [48], parafunctional habits like finger sucking, bruxism, nail-biting, and non-nutritional sucking were evaluated in a population of 403 6- to 12-year-old children. At least one parafunctional habit was found in 39% of participants. Bruxism was the most common, with 22%, followed by nail-biting (8.2%). This study showed a significant prevalence of parafunctional oral habits in pediatric patients [48]. These data show that the pediatric stage is a sensitive age range for dental and skeletal complications related to parafunctional habits, sometimes affecting the temporomandibular joint. In our study, eight (~31%) patients had previously received orthodontic treatment, two (~8%) had been submitted to wisdom teeth removal, and two (~8%) had suffered facial trauma. It is also important to note that four (~15%) patients had concomitant asthma. The association of some degree of inflammation in the respiratory system and the body seems to correlate with painful TMD. In a cross-sectional study published by Braido et al. [49], the authors described how bronchitis and asthma were statistically associated with painful TMD. Individuals affected by these two chronic respiratory inflammatory conditions presented an increased risk of 2.5 and 3.1 of having temporomandibular arthralgia. The connection seems to be an excessive use of the accessory respiratory muscles, with increased tension in the cervical region generating a nociceptive stimulus that triggers orofacial pain [49].

The present study’s limitations include: small sample size and the absence of a control group following conservative treatment. Without a control group (without interventions) and a conservative treatment group, we cannot evaluate whether this patient population would have improved with continued medical management and conservative treatments. It is also important in the future to consider an in-depth study of the risks of applying these techniques to the young population, as well as assessing the risks and benefits in terms of financial costs compared to conservative treatment.

## 5. Conclusions

TMJ arthrocentesis and arthroscopy can represent an effective and safe option to manage pediatric patients with arthrogenous TMD, integrating the armamentarium of available strategies and offering a minimally invasive, effective solution, especially when conservative treatment fails. We hope this research sheds light on this topic of interest, leading to more extensive studies and allowing clinicians to feel safer and more confident about performing these techniques in the pediatric population. Future research should compare therapeutic outcomes of TMJ arthrocentesis, arthroscopy, and continued medical management on a statistically significant level.

## Figures and Tables

**Table 1:** Criteria for intervention success.

Good	No pain or only mild pain level (VAS ≤ 2 on a 0–10 scale) and MMO ≥ 35 mm
Acceptable	No pain or only mild pain level (VAS ≤ 2 on a 0–10 scale) and MMO ≥ 30 mm and <35 mm
Failure	Constant or moderate pain (VAS > 2 on a 0–10 scale) and/or MMO < 30 mm

**Table 2:** Patient characteristics, clinical diagnosis, and treatments performed. DDwR: disc displacement with reduction; DDwoR: disc displacement without reduction; OA: osteoarthrosis; TMJ: temporomandibular joint.

Number of Patients	26	
Sex		Number of patients (%)
	Female	18 (69.2%)
	Male	8 (30.8%)
Mean age (mean ± SD)	14.81 ± 2.30 (10–17)	
Parafunctional habits		Number of patients (%)
	Awake bruxism	13 (50.0%)
	Sleep bruxism	7 (26.9%)
Past orofacial treatments/events		Number of patients (%)
	Orthodontic treatment	8 (30.77%)
	Wisdom teeth removal	2 (7.69%)
	Facial trauma	2 (7.69%)
Other comorbidities		Number of patients (%)
	Asthma	4 (15.39%)
	Allergic rhinitis	1 (3.85%)
	Depression	1 (3.85%)
Joints affected by arthrogenous disorder		Number of joints (%)
	Total	48 (92.31%)
	Right side only	0 (0%)
	Left side only	4 (15.38%)
	Bilateral	22 (84.62%)
Arthrogenous diagnosis		Number of joints (%)
	DDwoR + arthralgia	10 (20.83%)
	DDwR	9 (18.75%)
	Arthralgia	8 (16.67%)
	DDwR + arthralgia	7 (14.58%)
	DDwoR	4 (8.33%)
	DDwoR + OA	3 (6.25%)
	DDwoR + OA + arthralgia	2 (4.17%)
	DDwR + OA + arthralgia	2 (4.17%)
	DDwoR+ condylar resorption	1 (2.08%)
	DDwR + condylar resorption + arthralgia	1 (2.08%)
	DDwoR + disc perforation	1 (2.08%)
Myogenous diagnosis		Number of patients (%)
	Myalgia	18 (69.23%)
	I	3 (11.54%)
	II	7 (26.92%)
	III	8 (30.77%)
Treatment performed		Number of joints (%)
	TMJ arthrocentesis	32 (66.67%)
	TMJ arthroscopy	16 (33.33%)
Follow-up period (days)	419.2 ± 363.5 (31–1277 days)	

**Table 3:** Success rate of minimally invasive temporomandibular joint (TMJ) procedure.

Success Rate		
	A Single Minimally Invasive TMJ Surgery	Minimally Invasive TMJ Surgery + Additional Arthrocentesis
Good–acceptable	20 (75.92%)	22 (84.62%)
Failure	6 (23.08%)	4 (15.38%)

**Table 4:** Pre- and postoperative VAS pain scores, MMO, according to minimally invasive TMJ procedures (TMJ arthrocentesis and arthroscopy). *p*: *p*-value.

		VAS Pain				MMO			
Minimally Invasive TMJ Surgery	N Joints (%)	Preoperative VAS Pain, M ± SD	*p*	Postoperative VAS Pain, M ± SD	*p*	Preoperative MMO, M ± SD	*p*	Posoperative MMO, M ± SD	*p*
TMJ arthrocentesis	32 (66.67%)	4.04 ± 2.70	0.84	0.52 ± 1.65	0.89	35.65 ± 7.75	0.86	43.76 ± 5.11	0.45
TMJ arthroscopy	16 (33.33%)	3.76 ± 3.03		0.47 ± 1.38		37.56 ± 10.68		41.44 ± 4.93	

## Data Availability

Data are contained within the article.
